# H_2_O_2_-Assisted Fabrication of Stiff and Tough Hydrogel Using Natural Cysteine-Rich Protein

**DOI:** 10.3390/gels11121007

**Published:** 2025-12-13

**Authors:** Mengting Fan, Beizhe Huang, Yuhan Li, Ting Zhang, Ranjith Kumar Kankala, Jianting Zhang

**Affiliations:** 1 Institute of Biomaterials and Tissue Engineering, Huaqiao University, Xiamen 361021, China; 2 Fujian Provincial Key Laboratory of Biochemical Technology, Huaqiao University, Xiamen 361021, China

**Keywords:** protein hydrogel, protein unfolding, entanglement, disulfide bond, mechanical property

## Abstract

Protein-based hydrogels have emerged as an important class of materials for biomedical applications. However, these hydrogels often exhibit inferior mechanical properties, significantly limiting their potential applicability. Herein, a simplified yet efficient soaking method is developed to broaden the scope of constructing stiff and tough pure protein hydrogels, using natural cysteine-rich proteins, such as lactoferrin (LF). The preformed, soft and brittle, unfolded protein hydrogels transformed into highly stretchable and compressible elastomers after soaking in H_2_O_2_ due to chain entanglement and self-crosslinking via interchain disulfide bonds. As a result, the H_2_O_2_-treated LF hydrogels exhibited an extraordinary ultimate strength (compressive and tensile strains of over 90% and 400%, respectively, and stresses of 20 and 1.5 MPa). In addition, these rubber-like hydrogels exhibited exceptional self-recovery and fatigue resistance capabilities. Furthermore, the relationship between protein structure and the mechanical properties of the hydrogel was investigated. Together, these revelations could serve as a guiding principle for advancing the design of biocompatible, tough protein hydrogels without chemical modification or mechanical reinforcing fillers.

## 1. Introduction

Due to their notable attributes of high biocompatibility, biodegradability, and designability, protein-based hydrogels have attracted considerable attention from researchers for various applications, including but not limited to tissue engineering [1,2,3], drug delivery systems [4,5], and wound dressing [6,7], among others [8,9,10]. Typically, pure protein-derived hydrogels are softer and more fragile than synthetic polymeric gels, which exhibit substantial elasticity and toughness [11,12,13]. The discrepancy in these properties arises from the folded three-dimensional structure inherent to proteins, leading to the loss of the long-chain feature evident in polymers.

Indeed, most proteins contain hundreds of amino acids with modifiable functional groups. Therefore, the chain lengths of such proteins can theoretically extend to tens or even hundreds of nanometers when unfolded, resembling a chain-like “polymer”. This characteristic feature enables the construction of elastomeric protein hydrogels by exploiting the entanglement and crosslinking of the unfolded protein chains [14,15,16,17,18]. For example, Fu and colleagues used a de novo-designed ferredoxin-like protein to significantly enhance the stiffness of a hydrogel without inducing brittleness through chain unfolding/refolding, crosslinking, and entanglement [19]. This promising strategy of chain entanglement prevented protein hydrogels from becoming brittle while concurrently stiffening. The globulin proteins, such as bovine serum albumin (BSA), have also been successfully used to fabricate a highly stiff gel via heat-induced unfolding and chemical coupling [20]. Nevertheless, the design and construction of pure tough protein hydrogels remain challenging [21,22,23]. The relationship between intricate protein attributes, including molecular weight (or chain length), secondary structure, and functional groups, and the mechanical properties of resultant hydrogels remains unclear [24,25,26]. A comprehensive understanding of these inherent relationships is imperative for engineering protein hydrogels with tailored mechanical properties [27,28,29].

Herein, we present a simple yet highly efficient soaking method for fabricating a stiff and tough protein hydrogel using the cysteine-rich natural protein lactoferrin (LF) as the raw material. LF consists of 689 amino acids, including 34 cysteine residues, forming 17 disulfide bonds [30]. The unfolding of LF results in an approximately 230 nm chain with a notable concentration of active thiol groups. These long chains and high-density thiols induced LFs to form tightly intertwined networks of physical and chemical cross-linkage upon immersion in hydrogen peroxide (H_2_O_2_) solution. More importantly, the refolding of unfolded chains led to a volume shrinkage, resulting in a hydrogel with a negative swelling ratio and excellent mechanical properties, including high stiffness, toughness, and stretchability. The validity of this strategy was further corroborated by its successful application to other cysteine-rich proteins like BSA, OVA, and LZ. All the hydrogels formed from these proteins exhibited reinforced mechanical properties without resorting to chemical modifications or the incorporation of mechanical reinforcing fillers.

## 2. Results and Discussion

### 2.1. Preparation of Stiff and Tough LF Hydrogel

**Figure 1:**
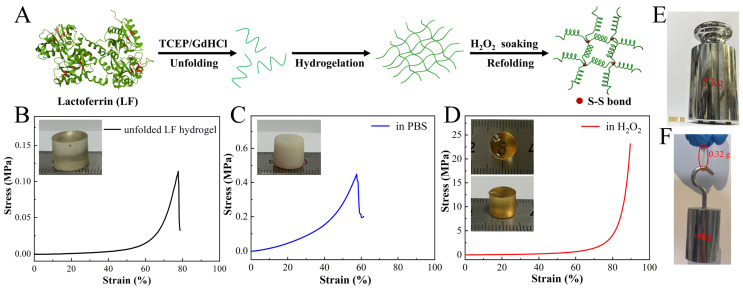
The formation of highly stiff and tough LF hydrogel via chain entanglement and self-crosslinking via disulfide bonds. (**A**) Schematic of the preparation process of LF hydrogel (Cys residues are shown in red). (**B**–**D**) Stress–strain curves and corresponding photographs of original unfolded LF hydrogel (**B**), hydrogel soaked in PBS (**C**), and hydrogel soaked in H_2_O_2_ (**D**) for 3 days. (**E**,**F**) Photographs of the LF hydrogel show that it can withstand a load of 5 kg without fracturing (**E**) and can carry more than 1500 times its weight.

As depicted in Figure 1A, LF proteins were denatured by the denaturant, guanidine hydrochloride (GdHCl), and the reducing agent of disulfide bond, tris(2-carboxyethyl)phosphine (TCEP), into a concentrated solution of unfolded chains. The resultant solution tended to undergo spontaneous transition to a soft hydrogel, particularly at concentrations exceeding 100 mg/mL (Figure S1). Chain overlaps and noncovalent interactions, such as hydrogen bonds, induced the hydrogelation of unfolded LF. The gelation time was found to depend on the pH value of the solution, which was consistent with reported protein-based hydrogels [30]. Under neutral pH conditions, the solution would quickly form a gel within a few minutes. We inferred that a decrease in electrostatic repulsion between unfolded chains could expedite gelation [31]. It should be noted that no disulfide bonds were formed in the hydrogel due to the presence of TCEP.

The unfolded LF hydrogel was immersed in the hydrogen peroxide (H_2_O_2_) for 3 days. It was observed that the H_2_O_2_-treated LF hydrogel exhibited distinctive macroscopic features compared to its original counterpart and controls equilibrated with PBS or ddH_2_O (Figure 1B–D). The PBS-equilibrated hydrogel displayed a shift towards opalescence and opacity, accompanied by a modest change in swelling index [16]. Conversely, the hydrogel soaked in ddH_2_O showed a substantial swelling ratio exceeding 600% due to the elevated osmotic pressure (Figure S2). In contrast, H_2_O_2_-treated LF hydrogel displayed a deep yellow color while retaining high transparency, allowing clear visibility of numerals beneath the hydrogel (Figure 1D). Notably, the hydrogel exhibited a remarkable negative swelling feature, with a swelling ratio of approximately −57%. This negative swelling phenomenon has been reported in the literature [19]. Due to the significant volume contraction of the hydrogel, it was challenging to detect pores in the freeze-dried sample from the scanning electron microscope (SEM) images (Figure S3). These conspicuous distinctions indicated a structural alteration in the unfolded LF chains upon exposure to H_2_O_2_ and PBS.

In the case of the hydrogel treated with PBS, the removal of the denaturant could trigger the LF refolding [16]. The refolding process led to relaxation of chain intertwining, resulting in the formation of a brittle and contractive gel. The compression test showed that the PBS-equilibrated LF hydrogel has greater stiffness than the original sample, but the failure strain decreased from 77% to 47%. For the H_2_O_2_-treated hydrogel, the free thiol groups within the hydrogel underwent rapid oxidation, forming interchain disulfide bonds that fastened the refolded LF chains. Consequently, the entanglement of chains was trapped within the covalent crosslinked hydrogel network. Further, chain refolding reinforced physical crosslinks, yielding a stiff and tough hydrogel. Circular dichroism (CD) spectra were used to analyze changes in LF chains. The characteristic peaks at 200 and 230 nm decreased dramatically after LF was treated with the denaturant. Subsequent dialysis into a 50 mM H_2_O_2_ solution obviously enhanced the CD intensity, indicating the refolding of unfolded LF chains (Figure S4A).

Further, these findings were confirmed by the Fourier-transform infrared spectroscopy (FTIR) analysis. In the FTIR spectra, the typical peaks at 1650–1655 cm^−1^ could be attributed to the amide I region (corresponding to α-helix), which were decreased after treating LF with denaturant (Figure S4B). Nevertheless, the peak corresponding to α-helix recovered after H_2_O_2_ soaking, indicating the refolding of α-helix [32]. Such a physically and chemically crosslinked hydrogel demonstrated amazing stiffness and toughness. As depicted in Figure 1D, the LF hydrogel demonstrated the ability to withstand a compressive strain exceeding 90% and a stress exceeding 20 MPa. Three such cylindrical LF hydrogels with a diameter of 8 mm and a height of 10 mm sustained a loading stress of 50 N without any fractures, bearing a load more than 1500 times their weight, and resisting slicing with a sharp knife (Figure 1E,F and Figure S5). To the best of our knowledge, the resultant LF hydrogel was superior to many reported protein hydrogels, such as gelatin or silk fibroin gel, which could only exhibit a kilopascal-order stiffness [33,34,35,36,37].

### 2.2. Highly Stretchable and Compressible LF Hydrogel

**Figure 2:**
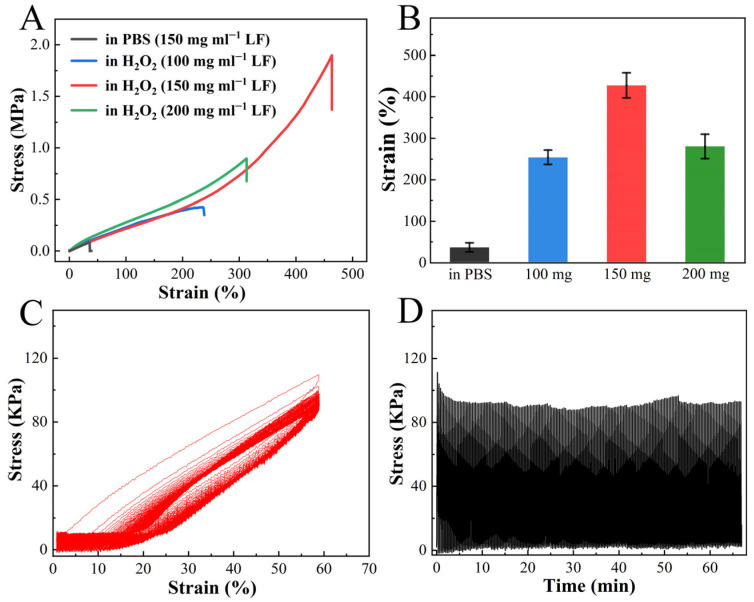
Tensile property of H_2_O_2_-treated unfolded LF hydrogel. (**A**) Tensile stress–strain curves of LF hydrogels with different concentrations after soaking in PBS or H_2_O_2_. (**B**) Fracture strain statistics for LF hydrogels with different concentrations after soaking in PBS or H_2_O_2_ (n = 5). (**C**,**D**) Consecutive loading-unloading curves (n = 200) of H_2_O_2_-treated LF hydrogel at a pulling speed of 100 mm min^−1^.

Remarkably, the tensile test revealed the exceptional stretchability of H_2_O_2_-treated LF hydrogel. As shown in Figure 2A, the fracture strain of the hydrogel prepared by 150 mg mL^−1^ LF was 426 ± 34% with a stress of 1.54 ± 0.36 MPa after H_2_O_2_ soaking. The resultant value was significantly higher than that of the PBS-equilibrated hydrogel, with a stress of 0.08 ± 0.02 MPa and a fracture strain of 35 ± 11% (Figure 2A,B). The original, unfolded hydrogel was soft and ruptured easily under slight stretching. An intriguing observation was made in terms of the correlation between the strength and stretchability of H_2_O_2_-treated LF hydrogel and the concentration of LF. It was observed that the hydrogel prepared by 150 mg mL^−1^ LF showed the highest breaking tensile strain compared to its 100 and 200 mg mL^−1^ counterparts (Figure 2B). The swelling test analysis revealed that LF hydrogels at different concentrations showed a similar volume shrinkage after H_2_O_2_ soaking (Figure S6A). It means these three hydrogels have different water content, leading to an obvious difference in stiffness (Figure S6B). In detail, the hydrogel at a concentration of 200 mg/mL presented the lowest water content but the highest stiffness. However, a dramatic increase in hydrogel stiffness is often accompanied by a compromise in stretchability [38]. Therefore, although the LF hydrogel at 200 mg/mL presented the highest stiffness, reaching a stress of ~5 MP at 80% strain, the stretchability of the LF hydrogel at 200 mg/mL was weaker than that of the LF hydrogel at 150 mg/mL. The H_2_O_2_-treated LF hydrogel was subjected to 200 consecutive loading-unloading cycles at a strain of 60%. The hydrogel showed little fatigue, retaining approximately 80% of the original stress in the first cycle, indicating a desired anti-fatigue performance (Figure 2C,D).

**Figure 3:**
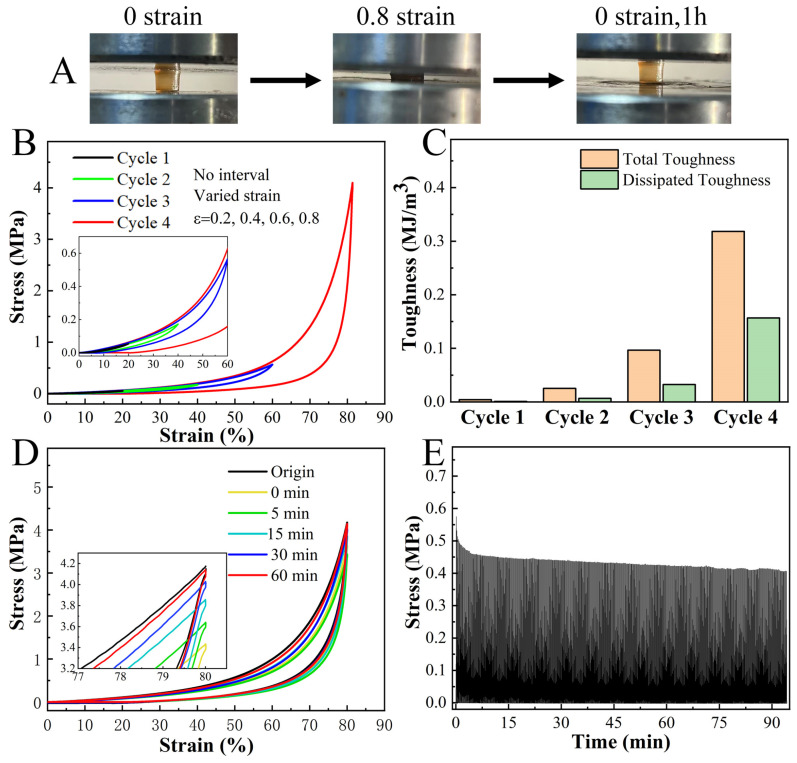
Self-recovery and fatigue resistance behavior of H_2_O_2_-treated LF hydrogels. (**A**) Photographs show that the LF hydrogel has a good property of fast recovery. (**B**) Sequential loading-unloading compression tests without interval and (**C**) the corresponding calculated total and dissipated toughness of the hydrogel under different strains (ε = 0.2, 0.4, 0.6, and 0.8). (**D**) Recovery cyclic compression tests show that the deformation of the N-DC hydrogel can be recovered to 80% of its applied strain. (**E**) 500 times consecutive loading-unloading curve of LF hydrogel at a pulling speed of 50 mm min^−1^. In each cycle, the hydrogel was stretched to 60% strain and subsequently relaxed to zero strain.

The H_2_O_2_-treated LF hydrogels not only possessed high compressive strength but also showed the excellent property of fast recovery (Figure 3A). In consecutive compression tests, the hydrogel promptly regained its original dimensions after unloading at lower strains (<60%), with small hysteresis (Figure 3B,C). At 80% strain, the hydrogel exhibited a noteworthy toughness of 320 KJm^−3^. About 80% of the original energy dissipation recovered immediately after unloading, with the remaining 20% recovering within 60 min (Figure 3D). Moreover, the hydrogel showed a desired anti-fatigue property, retaining about 75% of its original strength after 500 consecutive loading-unloading cycles at 60% strain (Figure 3E). Collectively, these results revealed that H_2_O_2_-treated LF hydrogels exhibited impressive features, including stiffness, toughness, and anti-fatigue performance. These characteristics are uncommon in protein hydrogels and even superior to those observed in certain polymer hydrogels [39,40].

### 2.3. Relationship Between Protein Structure and the Mechanical Properties of Hydrogel

**Figure 4:**
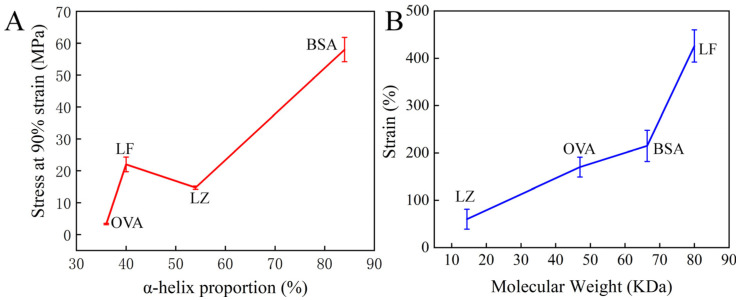
The relationship between protein attributes and the mechanical properties of hydrogel via examining the structure and properties of four cysteine-rich proteins and corresponding hydrogels. (**A**) *α*-helix proportion of four proteins and compression stress of the hydrogel made from the corresponding protein. (**B**) The molecular weight of four proteins and the tensile strain of the hydrogel made from the corresponding protein.

Further, this strategy was successfully extended to other cysteine-rich proteins, including bovine serum albumin (BSA), ovalbumin (OVA), and lysozyme (LZ). All of these proteins can form hydrogels based on our strategy (Figure S7). However, these resulting protein hydrogels exhibited distinct differences in the mechanical properties. Here, the relationship between the structural attributes of four proteins and the mechanical properties of their corresponding hydrogels was analyzed. As depicted in Table 1, the crystal structures of the four proteins and their respective attributes, including molecular weight, *α*-helix proportion, and thiol group density, are presented. The mechanical properties of four hydrogels were carefully investigated, including compression and tensile tests (Table 1). The resultant hydrogels exhibited pronounced negative swelling features, especially in the BSA hydrogel, which reached a ratio of −72 ± 2.6% (Figure S8). For the BSA hydrogel, volume shrinkage led to remarkable stiffness with a surprising compression stress of 58 ± 3.8 MPa at 90% strain, higher than that of the other hydrogels (Figure 4A). The high *α*-helix proportion and thiol density seem to play a significant role in the stiffness of the hydrogel prepared by our strategy [15,41]. This trend was corroborated by the OVA hydrogel, which had a low strength of 3.32 ± 0.21 MPa, reflecting its lower *α*-helix content and thiol density (Figure 4A).

In the tensile experiment, the stretchability of the hydrogel followed the order of LF > BSA > OVA > LZ (Table 1). The shortest LZ chain among these four proteins (~40 nm) has the highest density of thiol group (6.2%) for interchain covalent crosslinks [42]. However, LZ hydrogel exhibited a limited stretchability of less than 50% strain, merely one-eighth of that observed in LF hydrogel. We speculated that it was because the short LZ chains could not maintain a tight entanglement, resulting in a weak stretchability (Figure 4B). These results not only affirmed the efficacy of the H_2_O_2_ soaking strategy for constructing highly stiff and tough hydrogels from cysteine-rich proteins but also underscored the intimate relationship between protein structure and the mechanical properties of hydrogels. However, it is worth noting that the mechanical properties of protein hydrogel were also associated with other chemical attributes like hydrophobic interaction, hydrogen bond, among others, except for the molecular weight, *α*-helix proportion, and aforementioned thiol group density [20,23].

### 2.4. Biocompatibility Evaluation of LF Hydrogel

**Figure 5:**
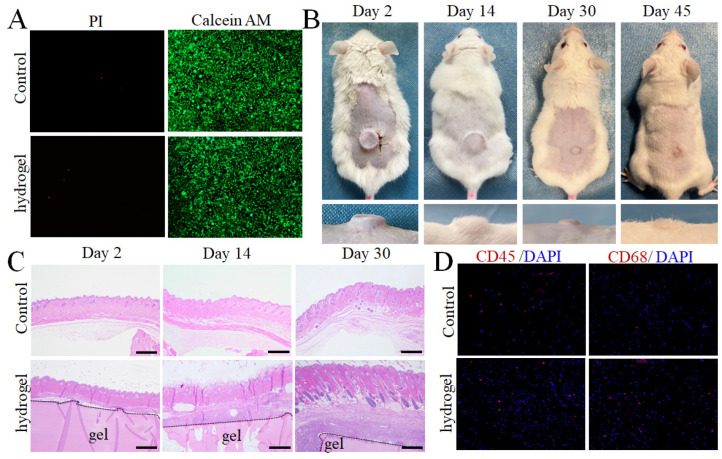
LF hydrogel shows high biocompatibility and biodegradability in vitro and in vivo. (**A**) Calcein-AM/PI fluorescence images of cells simultaneously stained with Calcein AM dye and propidium iodide (PI) for staining live and dead cells, respectively. (**B**) Macroscopic photographs of LF hydrogel implanted in subcutaneous tissue at different times. (**C**) H&E staining of LF hydrogel adjacent to skin tissues at 2, 7, and 30 days after subcutaneous implantation. (**D**) Immunofluorescence of LF hydrogel using CD45 and CD68 adjacent to skin tissues after 30 days of implantation.

The biocompatibility of LF hydrogel was further assessed in vitro and in vivo. Calcein-AM/PI co-stained fluorescence demonstrated high cell viability after incubation with leach solutions of LF hydrogel, indicating the low toxicity of these natural protein-based hydrogels (Figure 5A). A subcutaneous implantation experiment showed that the hydrogel also possesses biodegradation. In detail, the implanted hydrogel (1.5 mm × 7 mm) was gradually reduced and degraded completely after 45 days. The implanted LF hydrogel showed no significant effect on the mouse weight (Figure 5B and Figure S9). To evaluate inflammation induced by the LF hydrogel, the implanted hydrogel attached to the surrounding tissues was stained using H&E and toluidine blue (Figure 5C). The immunostaining assay results for CD45 and CD68 showed a low inflammatory response around the LF hydrogel after 48 h of subcutaneous implantation, further confirming the biocompatibility (Figure 5D). In addition, the major organs and hematological parameters were analyzed (Figures S10 and S11). These histological analyses revealed that hydrogel implantation did not elicit any significant inflammation around the hydrogel, further demonstrating the high biocompatibility of LF hydrogel. Therefore, these highly biocompatible, stiff and tough hydrogels could provide opportunities for potential biomedical applications that cannot be fulfilled by the soft hydrogels, such as repair of osteochondral defects [21].

## 3. Conclusions

We have demonstrated an H_2_O_2_-assisted strategy for fabricating a stiff and tough protein-based hydrogel by using the cysteine-rich protein as the primary raw material. Inspired by the network structure of elastic polymer hydrogels, cysteine-rich LFs were deliberately unfolded into long chains for physical entanglement and chemical crosslinking of interchain disulfide bonds induced by H_2_O_2_. The resulting hydrogels exhibited rubber-like characteristics, with compressive strain tolerance exceeding 90% with a stress of more than 20 MPa. Moreover, these hydrogels displayed a breaking tensile strain exceeding 400% with a remarkable toughness value of 1.3 MJ/m^3^. Such remarkable stretchability and load-bearing capacity were uncommon among reported protein hydrogels without chemical modification or the addition of mechanical reinforcing fillers. The mechanical properties of the protein hydrogel prepared based on our strategy have a close relationship with the features of the protein chain. The *α*-helix proportion and chain length significantly affected the stiffness and stretchability of the hydrogel. We believe this work may advance the design and construction of pure protein hydrogels with controllable mechanical properties.

## 4. Materials and Methods

### 4.1. Materials

All reagents were used as received without requiring further purification. Lactoferrin and BSA were purchased from Shanghai Aladdin Biotech. Co., Ltd., Shanghai, China. Guanidine hydrochloride (GdHCl, 99.5%), tris(2-carboxyethyl)phosphine (TCEP, 99%), lysozyme, and ovalbumin were acquired from Shanghai Sangon Biotech Co., Ltd., Shanghai, China. CCK-8 reagent kit and Calcein-AM/PI reagent kit were purchased from Shanghai Beyotime Biotechnology Co., Ltd., Shanghai, China. Paraformaldehyde, hematoxylin-eosin, and toluidine blue were purchased from Sigma-Aldrich Co., Ltd., St. Louis, MO, USA. The antibody was purchased from Abcam, Cambridge, UK.

### 4.2. Hydrogel Preparation

Proteins (LF, BSA, OVA, and LZ) were dissolved in ddH_2_O at a concentration of 200 mg mL^−1^. Subsequently, 0.67 g mL^−1^ GdHCl and 50 mg mL^−1^ TCEP were added, and the mixture was stirred to obtain an unfolded protein solution. The pH value of the solution was adjusted to 7.0 using NaOH solution. Further, the solution was poured into the silastic mold with a diameter of 12 mm and a height of 10 mm for gelation. The unfolded LF hydrogel formed within the silastic mold was carefully removed from the mold and immersed in PBS or PBS containing 50 mM H_2_O_2_ for 3 days. To ensure the complete removal of GdHCl and TCEP, the soaking buffer was changed daily.

### 4.3. Tensile Tests

Tensile tests were conducted employing a universal testing machine (MTS, Eden Prairie, MI, USA) equipped with a custom-made force gauge and a 100-N load transducer (MTS, Eden Prairie, MI, USA). Rectangular hydrogels were employed for tensile tests in ambient air at room temperature, with a pulling speed of 5 mm min^−1^. For the successive loading-unloading tests, the hydrogels were pulled at a rate of 100 mm min^−1^. The strain of the sample was calculated as the change in length relative to the initial length. The stress was determined by dividing the load by the initial cross-sectional area of the hydrogel sample. Toughness was quantified by integrating stress–strain curves as specimens were loaded to failure.

### 4.4. Compression Tests

Cylinder-shaped hydrogel compression tests were conducted employing the Instron-5500R (Instron, Norwood, MA, USA) equipped with a 5000-N load transducer in ambient air at room temperature. The sample was compressed and relaxed at a speed of 5 mm min^−1^. The compressive modulus was determined at a strain of 10–20%. Energy dissipation was quantified by integrating the loop area enclosed by compressing and relaxing stress–strain curves. For the successive loading-unloading tests, a compression speed of 50 mm min^−1^ was applied.

### 4.5. Volumetric Swelling Ratio Measurements

The dimensions of the initial cylindrical hydrogels were measured, and the volumes were recorded as V_i_. The swollen hydrogels V_s_ were recorded after 3 days of H_2_O_2_ soaking. The swelling ratio (r) was obtained from the following equation:

r = (V_i_ − V_s_)/V_i_

### 4.6. Scanning Electron Microscope Characterization

The hydrogel samples were prepared for SEM observation using a Hitachi S4700 scanning electron microscope (Hitachi, Tokyo, Japan). Firstly, samples were shock-frozen in liquid nitrogen and quickly transferred to a freeze-drier (Biocool, Beijing, China) for 24 h of lyophilization. Then, lyophilized samples were carefully fractured for SEM measurements.

### 4.7. Fourier Transform Infrared Spectroscopy Characterization

The changes in the amide bands of the LF hydrogel after H_2_O_2_ soaking were investigated using FTIR absorption bands obtained using a Bruker spectrometer (Bruker Optik GmbH, Ettlingen, Germany) in attenuated total reflection (ATR) mode. The freeze-dried samples were mixed with potassium bromide at a ratio of 1:100 and pressed into pellets for analysis. The scanning wavelength range was 4000–400 cm^−1^.

### 4.8. Circular Dichroism (CD) Characterization

The CD spectra of the protein solutions (0.1 mg/mL in PBS, 10 mM, pH 7.0) were obtained at 20 °C using a J-815 spectropolarimeter (Jasco, Tokyo, Japan) in a cell with 0.1 cm path length. For each protein solution, a new background was recorded with the corresponding protein-free buffer using the same GdHCl and TCEP or H_2_O_2_ concentrations. The CD spectra are presented as ellipticity normalized by a scaling factor.

### 4.9. In Vitro Biocompatibility

The biocompatibility of the LF hydrogel was analyzed using a Calcein-AM/PI staining and Cell Counting Kit (CCK)-8 assay (Beyotime, Shanghai, China). L929 cells were seeded in a 96-well plate at a density of 5 × 10^3^ cells per well overnight for proper cell attachment. Then, the hydrogel suspension was added to the corresponding wells with different concentrations. Then, the toxicity of the sample to 4T1 cells was further confirmed by co-staining with the Calcein-AM/PI assay kit (Beyotime, Shanghai, China).

### 4.10. In Vivo Experiments

The animal experiment was in strict adherence to the guidelines suggested for the Care and Use of Laboratory Animals. The Animal Ethics Committee of Huaqiao University approved all procedures involving animals in this study (A2023014). 5-week-old ICR male mice were randomly assigned to the test and control groups (n = 3). An incision (~10 mm) was made on the notum, and the customized hydrogel was implanted in the subcutaneous spaces of mice. The in vivo biocompatibility of LF hydrogel was confirmed by histological analysis. The tissue samples were fixed with 4% (*w*/*v*) paraformaldehyde (Sigma-Aldrich) and embedded in paraffin for sectioning (6 μm thick), which were stained with hematoxylin and eosin (H&E) and toluidine blue to assess the inflammatory response. The samples were also stained with primary antibodies against CD45 (1:100 dilution, Abcam, Cambridge, UK) and CD68 (1:100 dilution, Abcam, Cambridge, UK) and then immunofluorescently stained with Alexa 488- or 594-conjugated secondary antibodies (1:200 dilution, Invitrogen, Carlsbad, CA, USA). The counterstaining for cell nuclei was conducted with 4′,6-diamidino-2-phenylindole.

## Figures and Tables

**Table 1:** Structural properties of four proteins and their mechanical properties of the hydrogel made from the corresponding protein.

Protein	Attributes				Tensile		Compression	
	MW (kDa)	α-Helix Proportion (%)	Cysteine Number	Thiol Density (%)	Breaking Strain (%)	Breaking Stress (MPa)	Stress at 90% Strain (MPa)	Modulus *Y* (MPa)
LZ	14.4	54	8	6.2	60 ± 21	0.23 ± 0.06	14.7 ± 0.5	0.48 ± 0.03
OVA	47	36	6	1.5	170 ± 21	0.48 ± 0.11	3.3 ± 0.2	0.43 ± 0.04
BSA	66.4	84	35	6	215 ± 33	1.09 ± 0.16	58 ± 3.8	1.22 ± 0.11
LF	80	40	34	4.9	426 ± 34	1.54 ± 0.35	22 ± 2.3	0.72 ± 0.04

## Data Availability

The original contributions of the study are included in the article. Further inquiries can be directed to the corresponding authors.

## Supplementary Materials

The following supporting information can be downloaded at https://www.mdpi.com/article/10.3390/gels11121007/s1, Figure S1: Photograph of the behaviors of LF proteins in PBS (A), PBS + GdHCl (B) and PBS + GdHCl + TCEP (C); Figure S2: Photograph of unfolded LF hydrogel soaked in ddH_2_O; Figure S3: SEM characterization of LF hydrogels. (A–C) SEM images of original unfolded LF hydrogel which contain a mass of guanidine hydrochloride (A), LF hydrogel soaked in PBS (B), LF hydrogel soaked in H_2_O_2_ (C); Figure S4: Characterization of unfolding and refolding of LF chains. (A) CD measurements of native LF protein, unfolded LF and unfolded LF after H_2_O_2_ dialysis, (B) FTIR spectra of native LF protein, LF hydrogel before and after H_2_O_2_ soaking; Figure S5: Photographs show that the high toughness of LF hydrogel that can resist cutting with a sharp scalpel; Figure S6: (A) Photograph of LF hydrogel with different LF concentration after H_2_O_2_ soaking, (B) Stress-strain curves of the corresponding LF hydrogels; Figure S7: Photographs of other different protein hydrogels after H_2_O_2_ treatment; Figure S8: Swelling ratio of four hydrogels; Figure S9: Weight curves of mice after LF hydrogel implantation; Figure S10: H&E staining of major organs at 2 d and 30 d after implantation. Scale bars: 100 μm; Figure S11: Hematological parameters of mice at 30 d after implantation.
