# Supplementary material for: H2O2-Assisted Fabrication of Stiff and Tough Hydrogel Using Natural Cysteine-Rich Protein
Source: Gels. 2025 Dec 13;11(12):1007. doi: 10.3390/gels11121007 (PMC12732800; doi:10.3390/gels11121007)
Supplement: Supplementary file 1 [file gels-11-01007-s001.zip › gels-3999159-supplementary.pdf]

Supplementary Information

H_2O_2-Assisted Fabrication of Stiff and Tough Hydrogel Using Natural Cysteine-Rich Protein

*Mengting Fan, Beizhe Huang, Yuhan Li, Ting Zhang, Ranjith Kumar Kankala, Jianting Zhang**

Table of contents

S1. Characteristics of LF hydrogel

Figure S1. The behaviors of LF proteins treated with or without denaturant.

Figure S2. Photograph of unfolded LF hydrogel soaked in ddH_2O.

Figure S3. SEM characterization of LF hydrogels.

S2. The change in the secondary structure of LF

Figure S4. Characterization of unfolding and refolding of LF chains.

S3. Characterization of H2O2-treated protein hydrogel

Figure S5. Photographs show that the high toughness of LF hydrogel.

Figure S6. Stress-strain curves of H_2O_2-treated LF hydrogels made by different protein concentrations.

Figure S7. Photographs of H_2O_2-treated BSA, OVA and LZ hydrogels.

Figure S8. Swelling ratio of four hydrogels.

S3. Biocompatibility evaluation

Figure S9. Weight curves of mice

Figure S10. H&E staining

Figure S11. Hematological evaluation

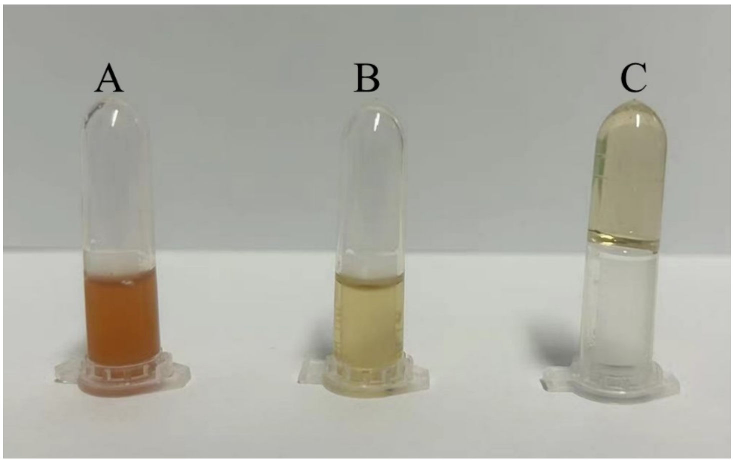

Figure S1. Photograph of the behaviors of LF proteins in PBS (A), PBS + GdHCl (B) and PBS + GdHCl + TCEP (C).

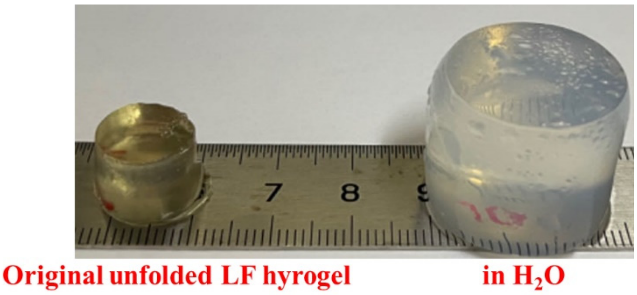

Figure S2. Photograph of unfolded LF hydrogel soaked in ddH_2O.

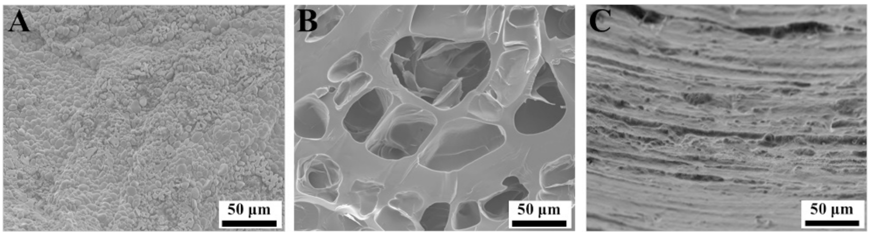

Figure S3. SEM characterization of LF hydrogels. (A-C) SEM images of original unfolded LF hydrogel which contain a mass of guanidine hydrochloride (A), LF hydrogel soaked in PBS (B), LF hydrogel soaked in H_2O_2 (C).

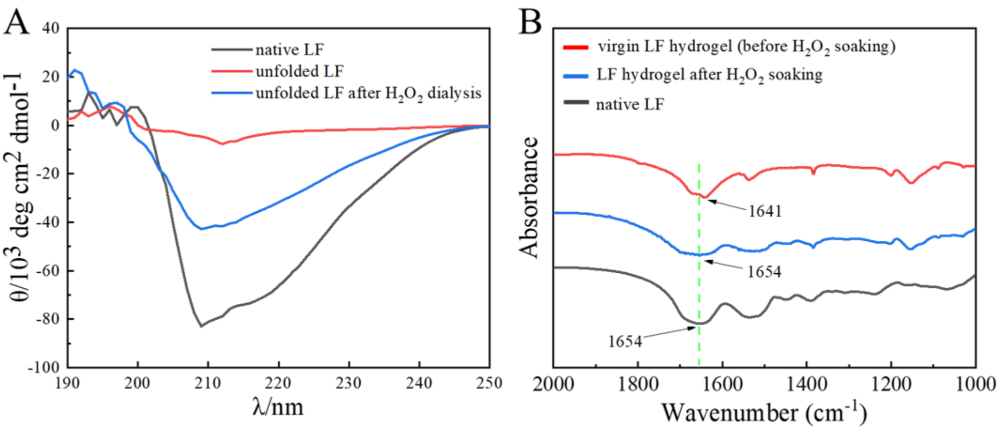

Figure S4. Characterization of unfolding and refolding of LF chains. (A) CD measurements of native LF protein, unfolded LF and unfolded LF after H_2O_2 dialysis, (B) FTIR spectra of native LF protein, LF hydrogel before and after H_2O_2 soaking.

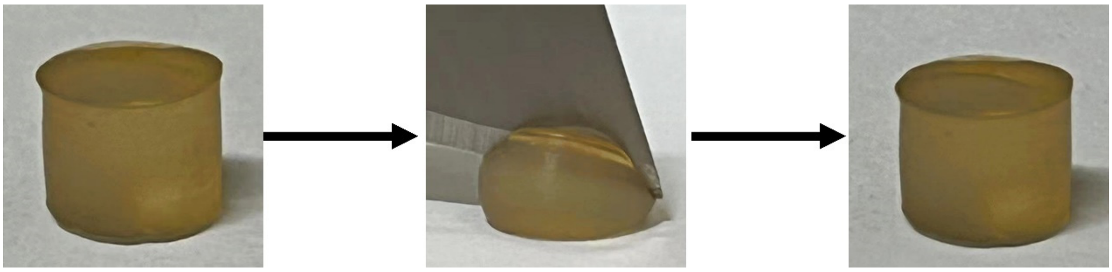

Figure S5. Photographs show that the high toughness of LF hydrogel that can resist cutting with a sharp scalpel.

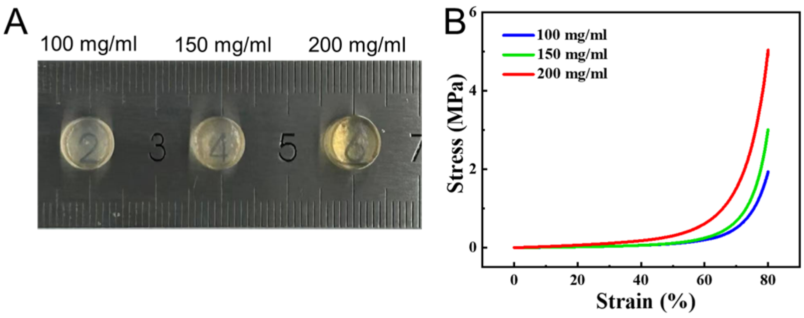

Figure S6. (A) Photograph of LF hydrogel with different LF concentration after H_2O_2 soaking, (B) Stress-strain curves of the corresponding LF hydrogels.

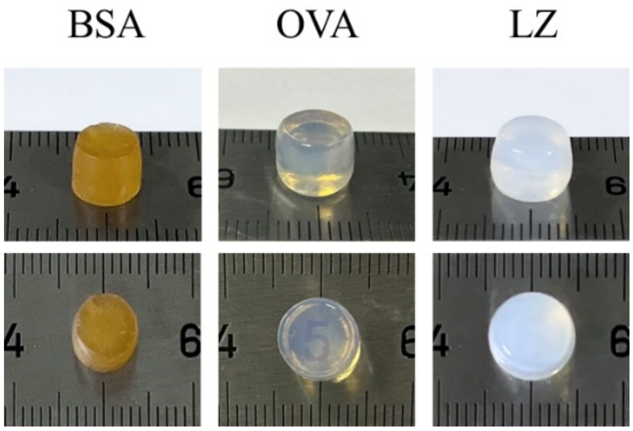

Figure S7. Photographs of other different protein hydrogels after H_2O_2 treatment.

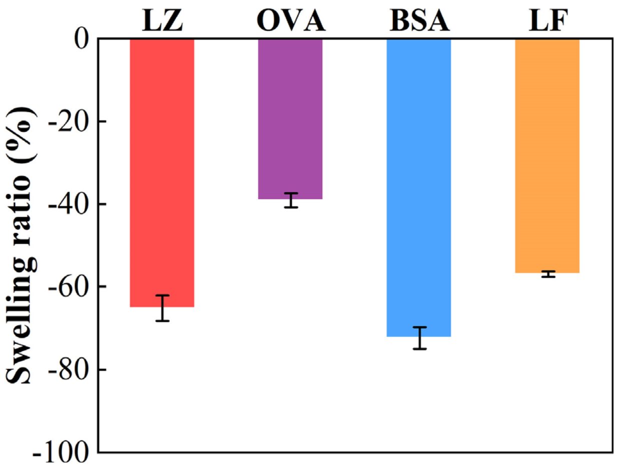

Figure S8. Swelling ratio of four hydrogels.

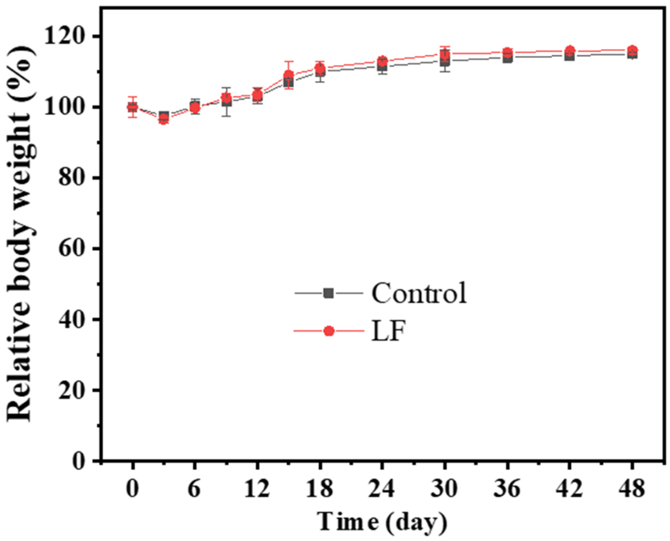

Figure S9. Weight curves of mice after LF hydrogel implantation.

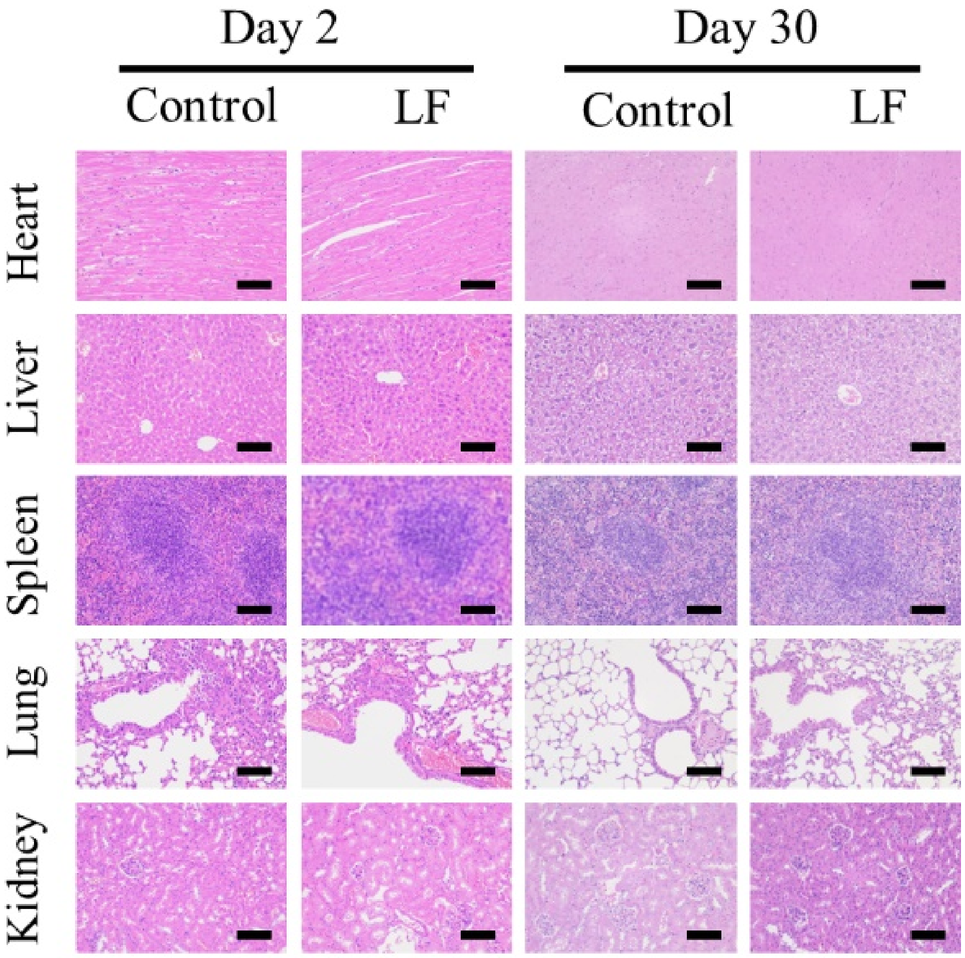

Figure S10. H&E staining of major organs at 2 d and 30 d after implantation. Scale bars: 100 μm.

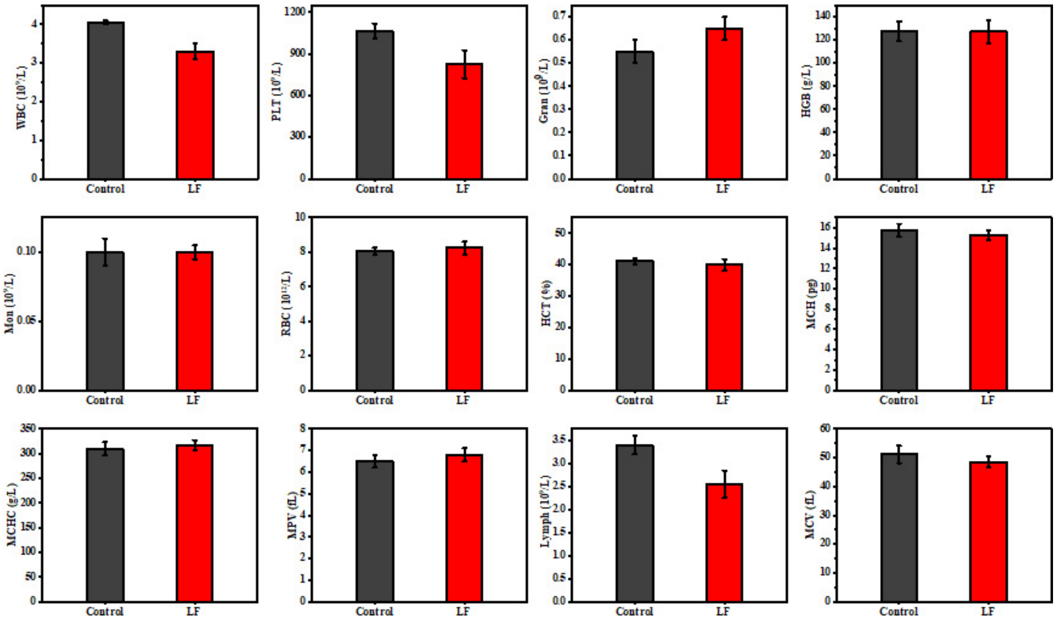

Figure S11. Hematological parameters of mice at 30 d after implantation.
